# Precision Agriculture Techniques and Practices: From Considerations to Applications

**DOI:** 10.3390/s19173796

**Published:** 2019-09-02

**Authors:** Uferah Shafi, Rafia Mumtaz, José García-Nieto, Syed Ali Hassan, Syed Ali Raza Zaidi, Naveed Iqbal

**Affiliations:** 1 National University of Science and Technology (NUST), School of Electrical Engineering and Computer Science, Islamabad 44000, Pakistan; 2 Department of Languages and Computer Sciences, Ada Byron Research Building, University of Málaga, 29016 Málaga, Spain; 3 School of Electronic and Electrical Engineering, University of Leeds, Leeds LS2 9JT, UK

**Keywords:** smart agriculture, precision agriculture, vegetation index, Internet of Things

## Abstract

Internet of Things (IoT)-based automation of agricultural events can change the agriculture sector from being static and manual to dynamic and smart, leading to enhanced production with reduced human efforts. Precision Agriculture (PA) along with Wireless Sensor Network (WSN) are the main drivers of automation in the agriculture domain. PA uses specific sensors and software to ensure that the crops receive exactly what they need to optimize productivity and sustainability. PA includes retrieving real data about the conditions of soil, crops and weather from the sensors deployed in the fields. High-resolution images of crops are obtained from satellite or air-borne platforms (manned or unmanned), which are further processed to extract information used to provide future decisions. In this paper, a review of near and remote sensor networks in the agriculture domain is presented along with several considerations and challenges. This survey includes wireless communication technologies, sensors, and wireless nodes used to assess the environmental behaviour, the platforms used to obtain spectral images of crops, the common vegetation indices used to analyse spectral images and applications of WSN in agriculture. As a proof of concept, we present a case study showing how WSN-based PA system can be implemented. We propose an IoT-based smart solution for crop health monitoring, which is comprised of two modules. The first module is a wireless sensor network-based system to monitor real-time crop health status. The second module uses a low altitude remote sensing platform to obtain multi-spectral imagery, which is further processed to classify healthy and unhealthy crops. We also highlight the results obtained using a case study and list the challenges and future directions based on our work.

## 1. Introduction

The rapidly-growing human population has increased food demands for human survival on the Earth. Meeting the food requirements with limited resources of the planet is a big challenge [1]. Several state-of-the-art technologies are being incorporated in the agriculture domain to enhance the productivity to cope with this challenge. Precision Agriculture (PA) is comprised of near and remote sensing techniques using IoT sensors, which help to monitor crop states at multiple growth levels. PA involves the acquisition and processing of a large amount of data related to crop health. Multiple parameters are involved in plants health, including water level, temperature and others. PA enables a farmer to know precisely what parameters are needed for healthy crop, where these parameters are needed and in what amount at a particular instance of time. This requires collecting massive information from different sources and different parts of the field such as soil nutrients, the presence of pests and weeds, chlorophyll content in plants and some weather conditions. All collected information needs to be analysed to produce agronomic recommendations. For instance, given the developmental stage of plants, their level of greenness (chlorophyll content) reveals the nutrients needed. This information is combined with the characteristics of the soil where the plant is located along with weather forecast. All collected information is further used to determine how much of a certain fertilizer should be applied to that plant on the next day. The delivery of agronomic information on the right time to farmer and ensuring that he/she applies these recommendations are key to enhancing the yields.

The foremost driver of PA is a WSN, which is a network of multiple wireless nodes connected together to monitor the physical parameters of environment. Each wireless node is comprised of a radio transceiver, a micro-controller, sensor(s), an antenna, along with other circuitry that enables it to communicate with some gateway to transmit information collected by the sensor(s) [2]. Sensors measure the physical parameters and send the collected information to the controller, which further transmits this information to the cloud or a portable device. The agriculture sector has multiple requirements comprised of soil statistics, crops’ nature, weather conditions, fertilizer types and water requirements. Crops have diverse requirements depending on different crops on the same land and the same plant on different lands with different weather conditions. Sensors monitor the varying behaviour of these crop parameters. Due to rapid advancement in WSN technologies, the size and the cost of sensors have reduced, which make it feasible to implement them in many sectors of life including agriculture. The most common sensors used in the agriculture domain that capture environmental parameters related to crops [3] are listed in Table 1.

In general, a WSN consists of one or more wireless nodes that are further connected with sensors. These nodes are tiny devices that are responsible for collecting data. Nodes are divided into two types, a source node that collects the data, and the other is sink or gateway node, which receives data from the source nodes. A sink node has more computational power compared to a source node. However, there are energy, memory, power, size, data rate and price constraints when choosing wireless nodes. Table 2 shows a comparison of wireless nodes along with their common specifications [3]. Among all wireless nodes presented in Table 2, MICA2 is considered to be more suitable as compared to others because of its large number of expansion connectors, which makes it suitable to connect with several sensors.

Many applications using WSN have been proposed since the last decade to monitor crops’ health remotely. In [4], a cyber-physical system was presented for monitoring of a potato crop. Cyber physical systems can be expressed as smart systems that are comprised of software, hardware and physical components, integrated together to sense the varying states of the real world. The proposed system consists of three layers: the first layer is the physical layer, in which all sensory data are collected; the second layer is the network layer in which data are transmitted to the cloud; the third layer is the decision layer in which the data are analysed and processed to make decisions according to the observations.

There are several challenges in IoT-based systems due to exponential increasing devices. As in a typical IoT network, every node transmits data to the remote cloud, which results in cloud congestion, and the main challenges underlying the IOT-based system are latency with minimum power requirements, better usage of bandwidth and intermittent Internet connectivity. Fog computing and edge computing are the state-of-the-art techniques to overcome these issues; which reduce the computational burden of cloud. The main goal of fog computing is to conserve energy and bandwidth, which helps to increase the quality of service to the end users. In [5], an energy-efficient architecture of the Fog of Everything was presented, which was comprised of six layers. The first layer was an Internet Of Everything (IOE) layer, where things (could be fixed, mobile or nomadic) functioned under multiple spatially-distributed clusters. The second was a wireless access network that supported Thing to Fog (T2F) and Fog to Thing (F2T) communication over the wireless channel. In the third layer, the connected fog nodes behaved such as a virtualized cluster. There was an inter-fog backbone in the fourth layer, which was responsible for connectivity among fog nodes. The next layer was the virtualization layer, which provided each connected thing with the ability to augment its limited resource set, exploiting the computation capability at the virtual clone. In the last layer, there was an overlay inter-clone virtual network that empowered Peer to Peer (P2P) communication. Then, a protocol stack for FOE was presented, which was further tested by creating a small prototype named as V-FOE and simulated on the iFogsim toolkit. The results provided strong evidence for the effectiveness of the proposed framework and more energy efficiency with reduced failure rate and delay.

The energy efficiency is the most serious consideration while developing any fog architecture. In [6], an energy-efficient protocol for a fog-supported wireless sensor network was presented, which maximized the lifetime of the network by uniformly distributing the energy among connected nodes. The performance of the proposed algorithm was compared with the existing state-of-the-art algorithms in MATLAB. The results showed that the proposed algorithm was highly energy efficient with a prolonged network lifetime.

Regardless of all the advancements in the IoT domain, the adoption of PA has been limited to some developed countries. Because of the lack of resources, remote sensing-based techniques to monitor crop health are not common in under-developed countries such as Pakistan, which results in a loss of yield. Pakistan is an agricultural country due to is large arable land and climatic variations, which make it suitable to cultivate multiple types of crops. Despite all these natural resources, Pakistan is still unable to produce massive yields [7]. The main reason behind the low production is traditional farming practices, which are used for crop health monitoring and yield estimation. These techniques are completely based on farmer’s intuition and experience. Farmers visit the fields in order to monitor the crop, which is very laborious and quite challenging in the case of large arable land. In this case, the area under insect/pest attack is inaccurately measured, which can result in over spraying of insecticide and pesticide, which adversely affects the nutrition in crops.

Keeping in mind all these issues, our motivation is to provide the industry and research communities with a survey of technologies, metrics and current practices concerning communication devices, sensors and platforms used to monitor and analyse multiple sources of data (spectral images, IoT, etc.) used in environmental and agriculture applications. As the main contribution, we generated a technological taxonomy for PA, which was driven by an IoT-based architecture to monitor the crops’ health. The developed system had two modules including wireless sensor network-based crop heath monitoring in which multiple sensors were used to get the real-time heath status of crop; the other one was NDVI-based analysis of spectra images captured by a drone to assess the chlorophyll content, which was further used to monitor the health of the crop.

The rest of the paper is organized as follows: Section 2 presents the most common wireless communication technologies used in the agriculture domain; Section 3 explains the spectral image-based remote sensing techniques, platforms and vegetation indices; Section 4 describes remote sensing applications in the agriculture sector; Section 5 presents a case study on IoT-based and UAV-based PA; Section 6 explains the experiments and results; challenges are discussed in Section 7; and conclusions and future directions are presented in Section 8.

## 2. Wireless Communication Technologies

Various communication protocols have been introduced in the last few decades due to the rapid increase in IoT devices and WSN technologies. Each protocol has its own specifications depending on the bandwidth, number of free channels, data rate, battery timing, price and other factors [8]. The most commonly-used protocols for wireless communication in IoT-based applications in agriculture are:

### 2.1. Cellular

Cellular technology is most suitable for applications that require an extraordinary data rate. It can utilize GSM, 3G and 4G cellular communication capabilities by providing reliable high-speed connectivity to the Internet, requiring higher power consumption. It requires infrastructure to be deployed and operation cost and back up staff for it with a centralized managed authority. 4G cellular technology requires more battery power, but cellular technology is a good option in underground wireless sensor networks, such as security systems in smart home projects and agriculture applications [9]. A smart irrigation systems was presented in [10], in which several soil moisture sensors were deployed in the field in the ZigBee mesh network. The reading captured from the fields were transmitted over the cloud using the cellular 4G LTE network.

### 2.2. 6LoWPAN

6LoWPAN is an IP-based communication protocol, which was the first protocol used for IoT communication. 6LoWPAN is also low cost because of the low bandwidth and low power consumption. 6LoWPAN supports multiple topologies such as star and mesh topologies. To handle interoperability between IPv6 and IEEE 802.15.4, there is an adaptation layer between the network layer and the MAC layer [8]. The applications for 6LoWPAN are monitoring the health equipment, environment monitoring and in security and home automation systems. In [11], a 6LoWPAN-enabled wireless sensor network was presented to monitor the soil properties of crops. The 6LoWPAN system architecture for precision agriculture application was discussed in [12] where the performance evaluation of this protocol was discussed with several baud rates and power constraints.

### 2.3. ZigBee

ZigBee is a wireless communication protocol widely used in precision agriculture to monitor environmental conditions related to crops’ health [13]. It is based on the wireless 802.15.4 standard. Basically, it was developed for personal area networks by the ZigBee alliance [8]. It has a flexible network structure, long battery life, supports mesh, star and tree topology with multi-hop data transmission, is easily installed and supports large nodes. It has a short range with limited data speed and is less secure compared to Wi-Fi-based systems. ZigBee is very common in smart agriculture applications such as smart green houses and smart irrigation systems [14]. In [15], a smart irrigation system was presented based on the ZigBee communication protocol. This system consisted of two nodes, i.e., a sensor node and an actuator node. The sensor node was comprised of soil moisture sensors, which monitored the water level in the soil. The actuator module was responsible for taking actions according to the water level of the soil. All communication was carried out by means of ZigBee protocol.

### 2.4. BLE

BLE is as famous as the Bluetooth smart technology, which is a suitable protocol for IoT applications including agriculture [16]. It is particularly designed for low bandwidth, low latency and short range for IoT applications. The main advantages of BLE over typical Bluetooth include lower setup time, lower power consumption and unlimited support for nodes in a star topology. It has a very limited range of 10 meters. However, the drawbacks are that it can only provide communication between two devices, it presents low security, and it can lose connection during communication. In [17], a BLE-based infrastructure was presented to collect the sensors’ data. The proposed system utilized a smart phone to collect the data of sensors using BLE, where sensors were deployed in the plants, i.e., soil moisture sensors and soil temperature sensor.

### 2.5. RFID

RFID systems consist of a reader and a transponder, which have a very small radio frequency, called the RF tag. This tag is programmed electronically with distinctive information that has a reading characteristic. RFID has two technologies for the tag system the first is the active reader tag system, and the other is the passive reader tag. Active reader tag systems are more expensive, as they utilize more battery power and use high frequencies. However, passive reader tag systems are low powered. Some IoT applications using RFID include smart shopping, healthcare, national security and smart agriculture applications. An IoT-based smart irrigation system based on RFID was presented in [18]. The system was comprised of soil moisture and soil temperature sensors along with a water control system, so it collected the reading of the sensors and sent these readings to the cloud using RFID communication protocols, where the user controlled a water pump based on the water level of the soil.

### 2.6. Wi-Fi

Wi-Fi is the most common communication protocol that enables devices to communicate over a wireless signal. Wi-Fi provides Wireless Local Area Network (WLAN) connectivity to millions of locations, i.e., homes, offices and public locations such as cafes, hotels and airports with high speed. The Wi-Fi protocol supports IEEE 802.11, 802.11a, 802.11b, 802.11g and 802.11n. Wi-Fi is widely used in IoT-based applications including agriculture systems, i.e., smart irrigation, crop health monitoring and greenhouses. In [19], an infrastructure was presented to monitor environmental parameters inside the greenhouse such as temperature, light intensity and soil moisture level. This platform was comprised of sensors that collected the data related to the environmental variation and sent to the cloud using Wi-Fi. Similarly, another smart agriculture system based on Wi-Fi communication protocols was presented in [20]. This last one consisted of a Raspberry Pi connected with multiple sensors, which collected the data. The collected data were further transmitted to the cloud using Wi-Fi communication protocols.

### 2.7. LoRaWAN

LoRaWAN operates on the LoRa network. LoRaWAN defines the system architecture and communication protocol of the network, while the physical layer of LoRa enables the link for long-range communication. LoRaWAN manages the frequencies in communication, data rate and power consumption for all devices. LoRaWAN is common in agricultural applications because of its large coverage area and low power consumption [21]. In [14], a smart irrigation system based on LoRaWAN was presented. Table 3 shows the comparison of all mentioned wireless communication protocols [8]. Among all wireless communication technologies, 6LoWPAN and ZigBee are considered to be more suitable for PA application because both are based on mesh networking, which makes them suitable to cover large area.

## 3. Spectral Image-Based Remote Sensing

Remote sensing has been widely used in PA to monitor crops’ health for the last two decades. Remote sensing is a phenomenon in which physical conditions of the Earth are observed remotely by calculating the emitted and reflected radiation from some distance. There are special cameras that are used to capture images for further analysis to find the characteristics of a specific area. Multiple platforms are used to mount these cameras that capture images of the objects.

### 3.1. Spectral Image Platforms

Remote sensing platform considerations for spectral images are airborne-based, satellite-based and Unmanned Aerial Vehicle (UAV)-based [22]. Each platform has its own coverage range, which is determined by three factors: (i) Ground Sampling Distance (GSD), which is computed in terms of spatial resolution, (ii) data collection rate or frequency and (iii) average distance between the object and sensor. Apart from coverage range, several factors [23] affect the performance of platforms, as mentioned in Table 4.

#### 3.1.1. Satellite-Based Platforms

Space-borne platforms for remote sensing are considered to be the most stable platforms among all others. These platforms consist of satellites, rockets and space shuttles. Space borne platforms are categorized based on the orbits and timing. The advantages of satellite-based remote sensing include high spatial resolution, which makes it promising to extract extensive time-series data. The images obtained by satellite platforms cover large area and are stable without noise, which is normally induced due to interference while image capturing. However, the main problem with satellite-based platforms is their high cost in the case of high spatial resolution images. The second problem is their strictly fixed time schedule, so data cannot be collected at critical timings. The re-visitation times vary from twice in one day to 16 days, depending on the orbit of the satellite. The other big problem is that satellite platforms are highly sensitive to weather conditions, so if the weather is cloudy, the captured image will have less detailed information. Table 5 shows the main types of satellites with their specifications [22].

Among all satellite platforms presented in Table 5, some satellite data are freely available, while others provide a commercial solution. The commercial solutions such as Pleiades-1 provide images with a high resolution and a revisit time of one day. QuickBird, Landsat-8 and Sentinel are frequently-used satellite platforms used to obtain hyperspectral imagery. QuickBird was launched in 2001 by USA. The Panchromatic (PAN) and four Multi-Spectral (MS) imagery sensors are used in QuickBird with a GSD of 0.7 × 0.7–2.6 × 2.6 m with a revisit time 1–3.5 days. QuickBird provides a small revisit time, but it is a commercial solution. In contrast to QuickBird, Landsat-8 and Sentinel provide free solutions. Landsat-8 was launched in 2013 by the USA. Landsat-8 provides a GSD of 16 days with PAN and 11-MS imagery sensors. Though revisit time of Landsat-8 is much higher compared to QuickBird, but it provides images with 11 different multi-spectral bands.

Sentinel is another broadly-used satellite launched by the EU. It currently has three missions, i.e., Sentinel-1, Sentinel-2 and Sentinel-3. These missions provide images with 21 MS bands with revisit times of 5–10 days depending on which Sentinel mission is used. However, Sentinel-2 is a commonly-used platform in precision agriculture as it provides data freely at a 10-m spatial resolution and covers a swath width of 290 km. By combining Sentinel-2A and Sentinel-2B, the revisit time is further reduced to five days, which helps in change detection. The complete details of all satellite platforms with their specifications are listed in Table 5, where other platforms such as SAT, MODIS and WordView are also considered.

#### 3.1.2. Airborne-Based Platforms

Airborne platform are flexible compared to satellite platforms, but still are expensive. The revisit time is in human control, which can be changed any time. The coverage area by this platform is much smaller than satellite-based ones, but relatively greater than the UAV platforms. Some common airborne platforms used for remote sensing [22] are given in Table 6.

#### 3.1.3. UAV-Based Platforms

UAV platforms are a vibrant alternative to satellite and airborne, which are quite flexible and cost effective. A typical UAV platform consists of a communication and navigation system that incorporates a set of sensors mounted on it. Among UAV platforms, there are mainly fixed-wing platforms. and multirotor options are available. The flying time is based on the payload weight. In general, a longer flying time is achieved by fixed-wing systems, which demands lighter weight payloads. For example, high-definition cameras weighing less than 300 grams as the payload of a fixed-wing UAV allow it to fly for around two hours using currently available battery power [24]. On the contrary, battery-powered multirotor UAV with higher payload capacity have a reduced flying time, i.e., around 15–25 min. Table 7 shows UAV platforms commonly used in the agriculture domain and concretely to monitor the health of crops remotely [22]. Among these platforms, DJI/Phantom-2 is a more suitable choice for intermediate agricultural land because of its low cost and ease of use. The other advantage of this platform is that it provides support for mounting multiple cameras, which helps to monitor the crop in multiple electro-magnetic bands. The American Aerospace/RS-16 is also an option because of its flight time and large are coverage, but due to its high cost, this platform is not common.

In [25], the ESAFLY A2500_WH helicopter was used to implement the platform of the UAV with Tetra cam ADC Micro as the camera to capture hyper-spectral images of two different types of cultivation, i.e., vineyard and tomato. The images captured by the UAV platform are very high in resolution, so more information can be extracted as compared to satellite images. To assess the health of a crop, three types of Vegetation Index (VI) maps have been computed.

### 3.2. Vegetation Indices

Using multi-spectral images from the remote sensors described above, a series of Vegetation Indices (VIs) can be computed. Vegetation Indices (VIs) obtained from remote sensing-based canopies are effective algorithms for quantitative and qualitative evaluations of vigour, vegetation cover and growth dynamics, among other applications [26]. Hitherto, no unified mathematical expression exists that defines all VIs due to the complexity of the several light spectra combinations, instrumentation, resolutions and platforms used. In particular, this section focuses on vegetation indices NDVI, GDVI and SAVI, as they are widely used in PA.

#### 3.2.1. NDVI

The Normalized Difference Vegetation Index (NDVI) is the most popular VI that is extensively used to find the content of green in PA applications [27,28]. It uses Red (R) and Near Infrared (NIR) channels to compute the NDVI index. More NIR light is absorbed by healthy vegetation; however, absorption ratio is very small for red light. NDVI is computed by Equation (1) and returns a value between −1 and 1 [29].

(1) $$\begin{array}{cc} NDVI & =\frac{NIR-R}{NIR+R} \end{array}$$

Higher NDVI value indicate healthy vegetation, while smaller values of NDVI show that vegetation is very small at that specific region. There is another form of NDVI, i.e., the Green Normalized Vegetation Index (GNDVI), which uses the green channel instead of red. GNDVI is computed by Equation (2):

(2) $$\begin{array}{cc} GNDVI & =\frac{NIR-G}{NIR+G} \end{array}$$

#### 3.2.2. Difference Vegetation Index

The DVI was proposed to reduce the effect of soil reflectance, which is not covered by NDVI [30]. DVI is different between the reflectance of the NIR band to the reflectance of the red band. DVI is also computed with the green band, i.e., GDVI. Both DVI and GDVI are computed by Equations (3) and (4):

(3) $$\begin{array}{cc} DVI & =NIR-R \end{array}$$

(4) $$\begin{array}{cc} GDVI & =NIR-G \end{array}$$

#### 3.2.3. SAVI

NDVI and DVI do not compensate the background effect of soil. Therefore, many vegetation indices were introduced to compensate the effect of soil reflectance. The Soil Adjusted Vegetation Index (SAVI), the Green Soil Adjusted vegetation Index (GSAVI), the Optimized Soil Adjusted Vegetation Index (OSAVI), the Green Optimized Soil Adjusted Vegetation Index (OGSAVI) and the Modified Soil Adjusted Vegetation Index (MSAVI) [31,32,33] are common among them, which are computed by Equations (5)–(9):

(5) $$\begin{array}{cc} SAVI & =\frac{1.5(NIR-R)}{\left(NIR+R+0.5\right)} \end{array}$$

(6) $$\begin{array}{cc} GSAVI & =\frac{1.5(NIR-G)}{NIR+G+0.5} \end{array}$$

(7) $$\begin{array}{cc} OSAVI & =\frac{\left(NIR-G\right)}{NIR+R+0.16} \end{array}$$

(8) $$\begin{array}{cc} GOSAVI & =\frac{\left(NIR-G\right)}{NIR+G+0.16} \end{array}$$

(9) $$\begin{array}{cc} MSAVI & =0.5[2\left(NIR+1\right)-\sqrt{{\left(2NIR+1\right)}^{2}-8\left(NIR-R\right)}] \end{array}$$

#### 3.2.4. NR and NG:

Normalized Red (NR) and Normalized Green (NG) are two other famous vegetation indices being used in PA [33]. NR focuses on the part of spectrum where radiation is absorbed by chlorophyll, while NG focuses on the part of the spectrum where radiation is absorbed by other pigments, excluding chlorophyll. NR and NG are computed by Equations (10) and (11):

(10) $$\begin{array}{cc} NR & =\frac{R}{NIR+R+G} \end{array}$$

(11) $$\begin{array}{cc} NG & =\frac{G}{NIR+R+G} \end{array}$$

## 4. Wireless Sensor Network Applications in Agriculture

Multiple applications of wireless sensor networks are being utilized today in the agriculture sector. Some very common applications are smart irrigation, smart fertilization, smart pest control and green house monitoring.

### 4.1. Smart Irrigation Systems

Smart irrigation is an artificial irrigation application that controls the quantity of water by making a decision about where water is needed. It is the most significant constituent in agriculture, which has a great impact on crops’ health, cost and productivity. One major aspect of smart irrigation is to avoid the wastage of water since most countries in the world are facing water scarcity problems. A smart irrigation system was presented in [34] in which a Raspberry Pi was used along with two sensors: a soil moisture sensor was used to assess the water level in the soil, while a temperature and humidity sensor was used to monitor the environmental condition. The Raspberry Pi was connected to these sensors and the water supply network. A mobile application was developed for remote monitoring and remote water flow control enabling both manual and automatic water flow control. In automatic mode, water flow was automatically turned ON/OFF based on the water level of the soil without human intervention. In manual mode, the user was able to monitor the soil moisture level. An alert was generated when the water level of soil was getting below a specific threshold, and the user turned it ON/OFF using a mobile application.

Power is a big concern in IoT-based platforms, so many researchers have developed power-efficient systems. A power-efficient water irrigation system was presented using solar power [35] in which the controller was connected to the soil sensor and water supply valve. The water valve was turned ON/OFF based on the water level monitored by the moisture sensor. The power was supplied by the solar panel, so the system was independent of any external power module. Another sensor-based IoT system for water irrigation was presented in [36] in which the controller controlled the opening and closing of a solenoid valve based on the water level of the soil. In addition, a series of weather alerts were sent to the user via a mobile application to update the temperature and humidity of the environment, which had a direct influence on the water level of the soil. In [37], an energy-efficient irrigation system for cultivated crops was presented using a wireless sensor network in which water was effectively controlled based on environmental conditions. This system estimated the quantity of water needed for normal irrigation based on the humidity, temperature and wind speed collected by sensors along with historical data.

In [38], an IoT-based irrigation system was presented using soil moisture sensors controlled by ATMEGA 328P on an Arduino UNO board along with a GPRS module. The data collected from the sensors were sent to the cloud, i.e., Things Speak, where graphs were generated to visualize the data trends. A web portal was also designed where the farmer was able to check the status of water, if it was ON/OFF. Similarly, a real-time prototype for an irrigation system was presented in [39] in which soil moisture sensors and soil temperature sensors were used to assess the water status of the soil. RFID was used to transmit data to the cloud for further data analysis. Using ATMEGA 328, a water sprinkler system for smart irrigation was presented in [40] using temperature, humidity and soil moisture sensors. The water sprinkler was controlled based on the soil moisture level to save water and reduce human effort. In [41], a cost-effective drip irrigation system for a home was proposed in which a Raspberry Pi, Arduino, electronic water control valve and relay were used. ZigBee protocols were used for communication. The user turned ON/OFF the water valve by sending commands to the Raspberry Pi, which further processed the commands through the Arduino.

The sensors placement is a big issue that affects the accuracy of sensors. A detailed discussion of soil moisture positioning in the field and their accuracy was presented in [42]. For real-time irrigation systems, complete software and hardware requirements, problems and challenges and advantages were discussed in [43] where a big picture of the complete system was provided.

### 4.2. Smart Fertilization System

Fertilizer is an artificial or natural substance having some chemical elements used to enhance the growth and productivity of plants. Manual spraying is a common technique used for fertilization. However, the optimal way of fertilization requires sensing capabilities to find the exact place where fertilizer is needed, which chemical components are missing and the amount of fertilizer needed. It is important to provide fertilizers in a very precise amount in order to improve productivity [44]. Multiple fertilization techniques have been presented by researchers since the last decade using WSN and IoT.

An automated fertilization system was presented in [45] using real-time sensors to measure the soil fertility. The system consisted of three modules including input, output and decision support. The decision support module measured the optimal amount of fertilizers needed for the growth of the plants based on the real-time sensory data captured by the sensors. A mechanical sensor named the “Pendulum Meter” was introduced in [46], which was used for optimal fertilization. This sensor was mounted on the tractor to measure the density of the crop, so the corresponding fertilizer spreader was controlled based on the readings of this sensor. The IEEE 802.11 Wi-Fi module was used for communication along with GPS. Real-time data of soil were collected by several sensors, i.e., soil moisture, temperature, conductivity, NO${}_{2}$, CO${}_{2}$, etc. A Geographical Information System (GIS) server was used to interpolate sensory data.

### 4.3. Smart Pest Control and Early Disease Detection System

Pest attacks are the root cause of low productivity in the agriculture sector. These pests result in several serious diseases in plants that affect the plant’s growth. However, disease prediction provides early warning to the farmers, which enables them to make appropriate decisions to control the disease on time. Pest control systems are comprised of electronic devices that enable humans to identify traps in a specific range of these electronic devices [47]. These electronic devices are sensors capable of calculating the environmental parameters for further analysis.

Much research has been done in the agriculture sector for early disease detection and pest control systems using more advanced and sophisticated technologies [48,49]. Multiple imagery sensors have been used by different researchers to collect imagery data, such as: RGB sensors, fluorescence imagery sensors, spectral sensors and thermal sensors [50]. The thermal sensors are used to measure the water status in the plant by measuring the temperature, since this parameter has a direct influence on the water level in the plants. RGB images have three colour channels, i.e., red, green and blue, which can be used to perceive the biometric effect in the plants. Multi- and hyper-spectral sensors capture images containing the spatial information of objects in multiple wavebands. The spatial resolution is dependent on the distance between the object and the sensor. That is why satellite images contain less spatial resolution as compared to low altitude platforms such as drones. The fluorescence sensors are used to distinguish the photosynthetic activities in the plants. Various image processing techniques are applied to these imagery data to identify the diseases in plants.

In [50], an IoT-based plants disease and pest prediction system was presented to minimize the excessive use of fungicides and insecticides. Weather condition monitoring sensors, i.e., temperature, dew, humidity and wind speed, are used to monitor weather parameters to find a correlation between pest growth with weather. The sensors have been deployed in orchards, and data collected from these sensors are sent to the cloud. The farmer is informed about the alarming condition of the pest attack on the crops.

From a different point of view, hyper-spectral images are used to analyse crops’ health and pest attack using manned or unmanned vehicles on which spectral cameras are mounted. The captured images are analysed in depth using machine learning techniques to identify the disease in the plants. Advance Neural Networks (ANNs) are more common for processing imagery data due to their ability to learn complex structures and patterns. Using hyper-spectral images, a system was presented [51] to identify disease or pest attack in crops. The proposed system for disease detection used an ANN with multiple layers.

Early disease detection in sugar beet plants was presented in [52]. For early detection, four supervised classification algorithms were applied on spectral images. Spectral images were then collected for each image, and multiple vegetation indices were calculated to be used in predictive and perspective analysis. The vegetation indices used were NVDI, SR, SIPI, PSSRaand PSSRb, ARI, REP, mCAIand RRE. These vegetation indices values were used as features in the dataset. Support Vector Machine (SVM), ANN, and decision tree were used for classification. A comparative analysis was performed which, showed that SVM outperformed other classifiers for disease detection with an accuracy of 97.12%. In [53], a data mining technique was applied on the already collected dataset of two types of crops, i.e., wheat and paddy (rice), in India. For dimension reduction, Sammon’s mapping was used for multi-dimension scaling, i.e., to reduce the dimension also for unsupervised learning. For high dimensional data, dimension reduction is required prior to performing further data analysis for better data visualization and accuracy, since redundant dimensions reduce the effectiveness of any data analysis algorithms. Principle Component Analysis (PCA) is a very often-used technique along with Sammon’s mapping. Data from multi-dimensions were reduced to two or three dimensions. Then, the Self-Organized Maps (SOM) algorithm for clustering was used to find correlations between the data. The accuracy comparison of SOM and Sammon’s mapping was presented, which showed that SOM performed better on a large dataset, while Sammon’s mapping was suitable for small ones. Smart phones played important role in data acquisition, which were further used to monitor the crops’ health. In [54], the health of wheat crop was monitored using near surface imagery captured by a smart phone. The crop was classified as healthy or unhealthy based on the green level by computing Gcc.

Most of the applications in PA have been either IoT-based in which multiple sensors are used to assess the health of the crop or remote sensing-based in which crop health is assessed by performing some computation on spectral images. We can compare crop health monitoring application based on some attributes such as which sensors are used in particular applications, whether web or mobile services are provided or not, etc. The comparative analysis of some existing crop health monitoring applications is presented in Table 8 based on some attributes.

To precisely monitor the crop health, both IoT-based techniques and remote sensing techniques should be used together to provide more reliable and accurate information about the crop. As a proof of concept, we present a case study in which a crop health monitoring system based on IoT and remote sensing techniques is proposed. We provide a complete end-to-end solution in the agriculture domain by facilitating the agricultural user with web and mobile services so that he/she could be informed about the latest condition of the crop in a timely manner. In this way, remedy actions could be performed in time, which will result in enhanced production.

## 5. A Case Study on UAV-Based and IoT-Based Precision Agriculture

**Figure 1:**
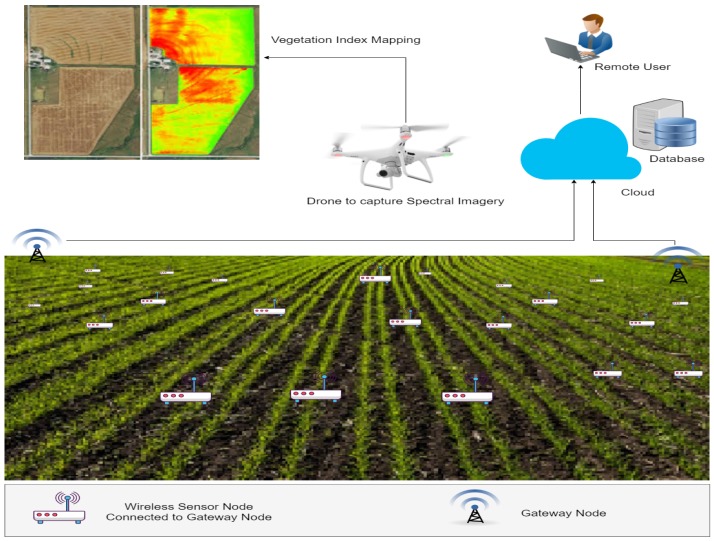
System architecture.

We developed a complete solution for crop health monitoring based on IoT and remote sensing. In the proposed system, crop health is monitored using data collected from multiple IoT sensors, as well as NDVI mapping of spectral images captured by a drone. The architecture of the proposed system is shown in Figure 1, which was designed according to two main modules. The first module was a wireless sensor network-based system in which multiple wireless nodes were developed. Each wireless node was comprised of a soil moisture sensor used to monitor the water level of the soil, a soil temperature sensor used to check the temperature of the soil and air temperature and humidity sensors. These nodes were deployed across the field in a star topology fashion where the master node collected readings from all slave nodes and transmitted the captured reading to the back-end server for further processing. The master node acted as a gateway node, which received data from all slave nodes using NRF communication module. After performing initial processing, the master node transmitted the data to the cloud using GSM communication technology. In the case of the unavailability of the GSM network, this node stored the captured data and transmitted to the cloud upon the availability of network.

The second module was used to monitor crop health using multi-spectral imagery, which was collected by a multi-spectral camera mounted on a drone. The NDVI was computed using Equation (1) to classify between healthy and unhealthy plants by measuring the chlorophyll content in the crops, which was further used to localize the area under stress precisely.

**Figure 2:**
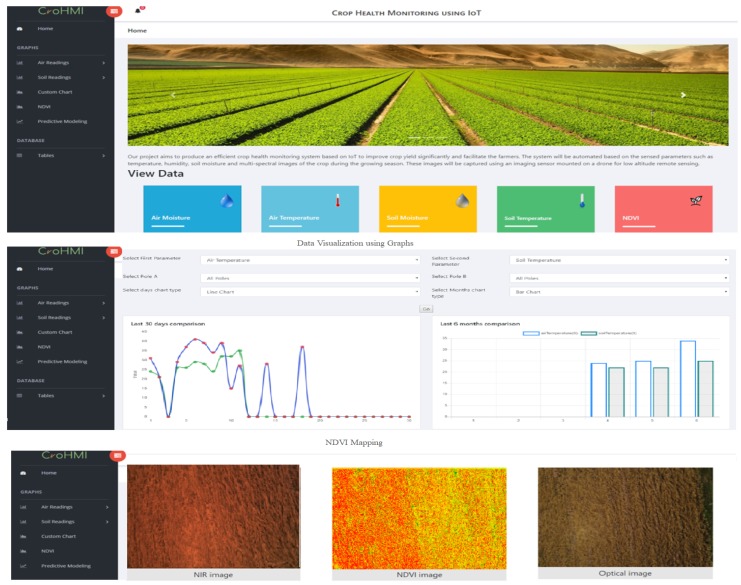
User interface of the web portal.

All collected data were sent to the cloud where further analysis was performed. The web portal was designed to help the farmer monitor the crop profile over the whole life cycle. Currently, we are monitoring soil moisture, soil temperature, air moisture and air temperature readings in real time along with NDVI mapping of spectral imagery. Multiple web services were provided on the web portal including historical/real data visualization using graphs, weather monitoring, NDVI mapping and the correlation among measured parameters. Figure 2 shows the snapshots of the web portal along with different services.

**Figure 3:**
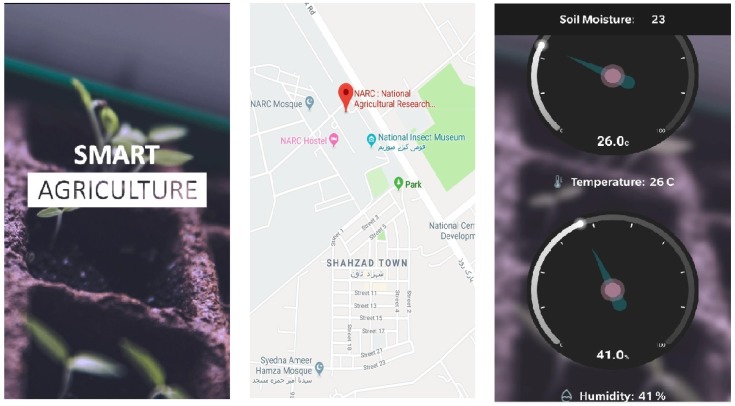
User interface of the mobile application.

For portability and remote monitoring, a mobile application was also developed to facilitate the farmer/agronomist/landlord with all the web services that are available on web portal. The alerts are generated when an abnormal behaviour is observed in the crop, which help the farmer to take remedy actions in a timely manner. The user interfaces of the mobile application are shown in Figure 3. Therefore, the web portal along with mobile applications provides a complete solution, which enables agricultural users monitor the current status of the crop, as well as previous details.

## 6. Results and Discussion

### 6.1. Analysis of the Data Collected by IoT Nodes

**Figure 4:**
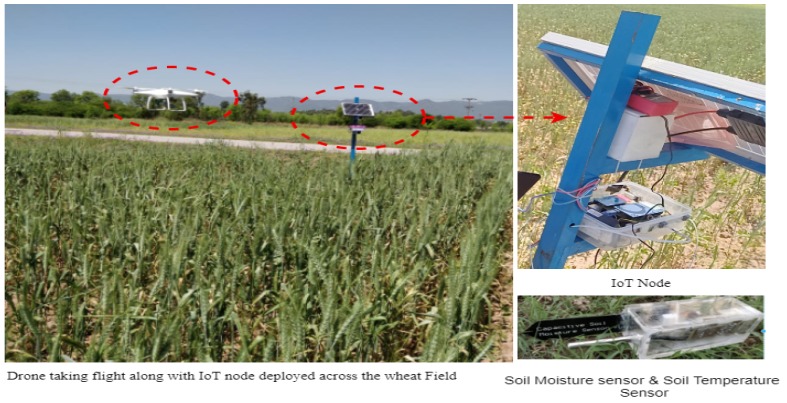
System deployed across the wheat fields.

The developed IoT nodes were deployed across the wheat fields of an area of 1.4375 hectare. The selected area was located in Islamabad, Pakistan. The wheat fields are shown in Figure 4 along with the IoT node and sensors. We deployed the system across the wheat field in March 2019 when wheat was in the grain filling and grain ripening stage.

**Figure 5:**
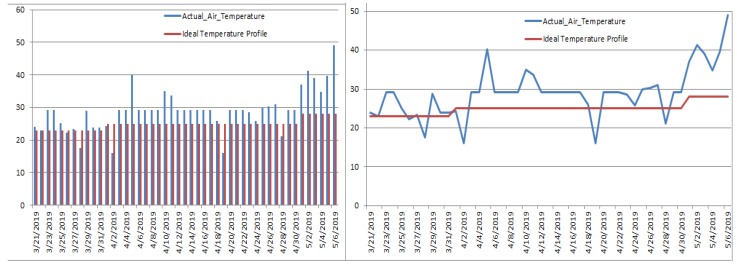
Deviation of observed temperature from the ideal temperature profile.

We collected the sensors’ readings such as air temperature, air humidity, soil temperature and soil moisture. We compared the observed crop parameters with the ideal wheat temperature profile as shown in Figure 5. Extreme variation in the weather of Islamabad was observed in that particular time period, which can be seen by how the actual temperature for wheat crop deviated from the ideal temperature profile of the wheat crop.

Additionally, we performed linear regression to find the correlation between observed parameters, which provided insight into how changes in one parameter can effect the other parameter. The linear regression found a relation between the two parameters by fitting the equation of the line using the observed dataset [69]. Equation (12) represents an equation of the line where mrepresents the slope of line, while c indicates the y-intercept. The variables m and c were learned from the data.

(12) y=mx+c

**Figure 6:**
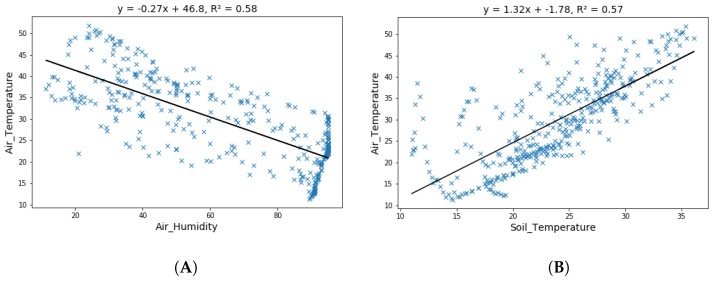
(**A**) Correlation b/wair temperature and air humidity. (**B**) Correlation b/w air temperature and soil temperature.

Figure 6A shows the correlation between the observed air temperature and air humidity, which showed that both were negatively correlated. The correlation between air temperature and soil temperature is shown in Figure 6B, which shows that both were positively correlated.

The rise in air temperature caused the air humidity to reduce, while it resulted in an increase in soil temperature and vice versa.

### 6.2. Analysis of Multi-spectral Images Captured by Drone

**Figure 7:**
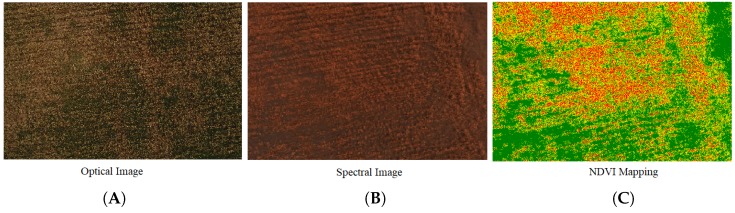
Wheat crop. (**A**) Optical image; (**B**) spectral image; (**C**) NDVI mapping.

To collect multi-spectral imagery, we used the DJI-Phantom Pro Advanced drone with the Sentera Multi-spectral-imaging sensor. The drone had its own optical camera, while the multi-spectral camera was mounted on it to obtain spectral images. Multiple flights of the drone were carried out at the specific growing stages of the crop, i.e., grain ripening and grain filling stage. After collecting these images, they were transferred to the cloud for NDVI mapping. Figure 7A shows the optical image that was captured on 16 May 2019 when wheat was in the harvesting stage, while Figure 7B is its spectral image, and Figure 7C is the NDVI mapping. Since wheat was at a mature stage, its NDVI should be very small, i.e., ideally there should be no green region in the field. However, in NDVI mapping, a large green region indicated the abnormal behaviour of the crop. The green region was due to naturally growing plants. This information can be visualized on the web portal.

**Figure 8:**
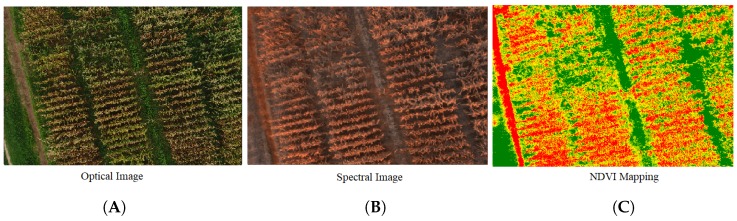
Maize crop. (**A**) Optical image; (**B**) spectral image; (**C**) NDVI mapping.

The same process was performed in a maize field when maize was in the grain ripening stage. Figure 8A shows the optical image that was captured on 24 July 2019 when maize was in the grain ripening stage, while Figure 8B is its spectral image, and Figure 8C is the NDVI mapping. The same behaviour can be observed with the maize crop, i.e., there should be a minimal green region in the field. However, in NDVI mapping, a large green region indicated the abnormal behaviour of the crop.

## 7. Challenges

PA has been used since the last few decades to enhance crops’ yield with reduced costs and human effort, although the adoption of these novel techniques by farmers is still very limited owing to the following reasons or challenges.

### 7.1. Hardware Cost

PA relies mostly on hardware such as sensors, wireless nodes, drones, spectral imaging sensors, etc., which are used to assess multiple parameters in real time. These sensors have multiple limitations including high development, maintenance and deployment cost. Some systems in PA are cost effective and are suitable for small arable land, i.e., smart irrigation systems that require low-cost hardware components and sensors. However, drone-based systems for crops’ health monitoring are feasible for large arable land due to high installation cost.

### 7.2. Weather Variations

Environmental variation is one of the major challenges that affects the accuracy of data collected by sensors. Sensor nodes deployed in the field are sensitive to environmental variations, i.e., rain, fluctuation in temperature, wind speed, sun light, etc. Communication between wireless nodes and the cloud can be interrupted due interference induced in wireless communication channels by atmospheric disturbance. The satellite, air borne and drone platforms are also sensitive to weather variations. Imagery acquired by these platforms is affected by contamination of clouds and other natural aerosols. The development of advanced techniques for atmospheric correction, cloud detection and noise interpolation is a current open challenge, which requires hard efforts from the research community.

### 7.3. Data Management

The sensors in PA constantly generate data. To ensure the integrity of data, some data security measures needs to be in place, which will in turn enhance the cost of the system. The readings from the sensors have to be accurate in order to take appropriate actions precisely when and where required. An intruder can corrupt the readings, and false readings will adversely reduce the effectiveness of the system. PA systems generate immense amounts of data, which require enough resources to perform data analysis. Real-time data collected from sensors deployed across the fields after a few minutes and spectral imagery acquired from high-altitude or low-altitude platforms produce the bulk of the data, which increase the storage and processing requirements. New software platforms and facilities for scalable management of Big Data sources are demanded. In this regard, the generation of software-as-a-service solutions is focused on merging data management and IoT thorough cloud computing platforms.

### 7.4. Literacy Rate

Literacy is an important factor that influences the adoption ratio in PA. In developing countries where the illiteracy rate is high, farmers grow crops based on their experience. They do not utilize the state-of-the-art technologies in agriculture, which results in loss of production. Farmers need to be educated in order to understand the technology or they have to trust a third party for technical support. Therefore, in underdeveloped areas where the literacy rate is not high, PA is not very common due to the limitations of resources and education.

### 7.5. Connectivity

Next-generation 5G networks can be 100-times faster than 4G ones, making communication between devices and servers much quicker. 5G can also carry much more data than other networks, which makes it an ideal technology for transmitting information from remote sensors and drones, key tools that are being tested in PA environments. The adoption of new communication networks based on 5G is a must in current applications where secure and rapid data transfer enables real-time data management and support for decision making.

### 7.6. Interoperability

One of the biggest problems PA faces is the interoperability of equipment due to different digital standards. This lack of interoperability is not only obstructing the adoption of new IoT technologies and slowing down their growth, but it also inhibits the gain of production efficiency through smart agriculture applications. New methods and protocols to integrate different machine communication standards to unlock the potential of efficient machine-to-machine communication and data sharing between machines and management information systems are required in the current scenario of PA.

## 8. Conclusion and Future Directions

Precision agriculture is a modern practice used to enhance crops’ productivity using latest technologies, i.e., WSN, IoT, cloud computing, Artificial Intelligence (AI) and Machine Learning (ML). Most of the research done so far indicates that PA-based practices have a great influence on sustainability and productivity. The objective of PA is to provide decision support systems based on multiple parameters of crops, i.e., soil nutrients, water level of the soil, wind speed, intensity of sunlight, temperature, humidity, chlorophyll content, etc. However, several challenges are involved in the development and deployment phase of these systems. This article was aimed at providing a survey of modern technologies involving current PA platforms, with the goal of supporting industry and research communities on the development of modern applications for smart agriculture. A case study was presented to prove the effectiveness of the PA in the agriculture domain.

Since the main objective of precision agriculture is to produce surplus yield by optimizing the resources such as water, pesticides, fertilizers, etc., for resource optimization, prescription maps play an important role, which enables farmers to quantify resources required for healthy crops at any particular growth stage. Most of the research accomplished in the agriculture domain focuses on the remote sensing platforms to collect imagery, which reflects only Vegetation Indices (VIs) such as NDVI. The prescription maps cannot be generated by only using VIs; instead, multiple other factors need to be considered such as soil properties, soil moisture level, meteorological behaviour, etc.

## Figures and Tables

**Table 1:** Wireless nodes used in the agriculture domain.

Sr #	Sensor Name	Parameters Captured
1	ECH2O soil moisture sensor	Soil Temperature, Soil Moisture, Conductivity
2	Hydra probe II soil sensor	Soil Temperature, Salinity level, Soil Moisture, Conductivity
3	MP406 Soil moisture sensor	Soil Temperature, Soil Moisture
4	EC sensor (EC250)	Soil Temperature, Salinity level, Soil Moisture, Conductivity
5	Pogo portable soil sensor	Soil Temperature, Soil Moisture
6	107-L temperature Sensor (BetaTherm 100K6A1B	Plant Temperature
	Thermistor)	
7	237 leaf wetness sensor	Plant Moisture, Plant Wetness, Plant Temperature
8	SenseH2™ hydrogen sensor	Hydrogen, Plant Wetness, CO${}_{2}$, Plant Temperature
9	Field scout CM1000TM	Photosynthesis
10	YSI 6025 chlorophyll sensor	Photosynthesis
11	LW100, leaf wetness sensor	Plant Moisture, Plant Wetness, Plant Temperature
12	TT4 multi-sensor thermocouple	Plant Moisture, Plant Temperature
13	TPS-2 portable photosynthesis	Photosynthesis, Plant Moisture, CO${}_{2}$,
14	LT-2 M (leaf temperature sensor)	Plant Temperature
15	PTM-48A photosynthesis monitor	Photosynthesis, Plant Moisture, Plant Wetness, CO${}_{2}$, Plant Temperature
16	Cl-340 hand-held photosynthesis	Photosynthesis, Plant Moisture, Plant Wetness, CO${}_{2}$, Plant Temperature, Hydrogen level in Plant
17	CM-100 Compact Weather Sensor	Air Temperature, Air Humidity, Wind Speed, Air Pressure
18	HMP45C (Vaisala’s HUMICAP® H-chip)	Air Temperature, Air Humidity, Air Pressure
19	Met Station One (MSO)	Air Humidity, Air Temperature, Wind Speed, Air Pressure
20	XFAM-115KPASR	Air Temperature, Air Pressure, Air Humidity
21	SHT71, SHT75 (Humidity and temperature sensor)	Humidity and Temperature Sensor
22	107-L Temperature Sensor (BetaTherm 100K6A1B thermistor)	Air Temperature
23	Cl-340 hand-held photosynthesis	Air Temperature, Air Humidity

**Table 2:** Common wireless nodes used in the agriculture domain.

Sr #	WN^1^	MC^2^	Expansion Connector	Available Sensors	Data Rate
1	MICA2DOT	ATmega128L	19 Pins	GPS, Light, Humidity, Barometric Pressure, Temperature, Accelerometer, Acoustic, RH	38.4 K Baud
2	Imote2	Marvell/XScalePXA271	40 Pins and 20 Pins	Light, Temperature, Humidity, Accelerometer	250 Kbps
3	IRIS	ATmega128L	51 Pins	Light, Barometric Pressure, RH, Acoustic, Acceleration/ Seismic, Magnetic and Video	250 Kbps
4	MICAz	ATmega128L	51 Pins	Light, Humidity, Temperature, Barometric Pressure, GPS, RH, Accelerometer, Acoustic, Video Sensor, Sounder, Magnetometer, Microphone	250 Kbps
5	TelosB	TIMSP430	6 Pins and 10 Pins	Light, Temperature, Humidity	250 Kbps
6	Cricket	ATmega128L	51 Pins	Accelerometer, Light, Temperature, Humidity, GPS, RH, Acoustic, Barometric Pressure, Ultrasonic, Video Sensor, Microphone, Magnetometer, Sounder	38.4 K Baud
7	MICA2	ATmega128L	51 Pin	Temperature, Light, Humidity, Accelerometer, GPS, Barometric Pressure, RH, Acoustic, Sounder, Video, Magnetometer	38.4 K Baud

WN^1^: wireless node, MC^2^: Micro-Controller.

**Table 3:** Wireless communication protocols used in Precision Agriculture (PA).

Communication Protocols	Data Rate	Topology	Standard	Physical Range	Power
6LoWPAN	0.3–50 Kb/s	Star, Mesh	IEEE 802.15.4	2–5 km urban,15 km suburban	Low
ZigBee	250 Kb/s	Star, Mesh Cluster	IEEE 802.15.4	10–100 m	Low
Bluetooth	1–2 Mb/s	Star, Bus	IEEE 802.15.1	30 m	Low
RFID	50 tags/s	P2P	RFID	10–20 cm	Ultra low
LoRa WaAN	27–50 Kb/s	P2P, Star	IEEE 802.11ah	5–10 km	Very low
Wi-Fi	1–54 Mb/s	Star	IEEE 802.11	50 m	Medium

**Table 4:** Key differences between spectral image platforms.

Applicability Aspect	Airborne	UAV	Satellite
Observation Area	Regional	Local	Worldwide
Ground Coverage	1 km (Medium)	100 m (Small)	10 km (Large)
Field of View	Wider	Wide	Narrow
GSD (Spatial Resolution)	5–25 cm	10–5 cm	0.30–300 m
Deployability	Complex	Easy	Difficult
Spatial Accuracy	1–25 cm	5–10 cm	1–3 m
Repeat Time	Hour(s)	Minute(s)	Day(s)
Operational Risk	High	Low	Moderate

**Table 5:** Satellite platforms for RS.

Name	Launch	Sensor	Country	Swath Width (km)	GSD ^1^ Range (m)	Revisit Time (day)
RapidEye	2008	5 MS^2^	Germany	77	6.5 × 6.5	1–5.5
QuickBird-2	2001	PAN^3^	USA	16.8–18	0.7 × 0.7	1–3.5
		4 MS^2^			2.6 × 2.6	
Pleiades 1	2011	PAN^3^	France	20	0.5 × 0.5	1
	2012	4 MS^2^			2 × 2	
Sentinel-1	2014	C-band	EU	80	5 × 5	12
	2016	SAR^6^		250	5 × 20	6 (dual)
				400	25 × 40	
WorldView-3	2014	PAN^3^, 8 MS^2^, 8 MS^2^	USA	13.1	0.3 × 0.3	1–4.5
		(SWIR^4^), 12 MS^2^			1.2 × 1.2	
					3.7 × 3.7	
Landsat-8	2013	PAN^3^, 11 MS^2^	USA	185	15 × 15	16
					30 × 30	
Sentinel-2	2015	13 MS^2^	EU	290	10 × 10	10
					20 × 20	
	2016				60 × 60	5 (dual)
EnMap	2017	232 HSI^5^	Germany	30	30 × 30	4
ICESat	2003	2 HSI^5^	USA	N/A	70	8
					(footprint)	
TanDEM-X	2007	X-band	Germany	5 × 10	1 × 1	11
		SAR^6^		1500 × 30	3 × 3	
				1500 × 100	16 × 16	
SkySat	2013	PAN^3^	USA	2 × 1	1.1 × 1.1	0.5 (2015)
		Video				
	2014	PAN^3^		8	0.9 × 0.9	0.12 (2017)
	2015	4 MS^2^			2 × 2	
ICESat-2	2018	1 HSI^5^	USA	N/A	10	N/A
		(9-beam)			(footprint)	
Sentinel-3	2015	21 MS^2^	EU	1270	300 × 300	0.25
	2017	11 MS^2^		1420	500 × 500	
		(IR)		750 (nadir)	1000 × 1000	
RADARSAT-2	2007	C-band	Canada	20	3 × 3	24 (orbit repetition)
		SAR^6^		500	100 × 100	
SPOT 6	2012	PAN^3^	France	60	1.5 × 1.5	1–5
SPOT 7	2014	4 MS^2^			6 × 6	
TerraSAR-X	2007	X-band	Germany	5 × 10	1 × 1	11
		SAR^6^		1500 × 100	16 × 16	
DMC-3	2015	PAN^3^	U.K.	23	1 × 1	1
		4 MS^2^			4 × 4	

GSD^1^: Ground Sampling Rate, MS^2^: Multi-Spectral, PAN^3^: Panchromatic, SWIR^4^: Short Wave Infrared, HSI^5^: Hyperspectral Imagery, SAR^6^: Synthetic Aperture Radar

**Table 6:** Airborne/aircraft platforms for RS.

Aircraft Type	Typical Models	RS Sensors	Max Flying Height (ft)
Fixed wing (jet)	LearJet 35A	InSAR,	45,000
		Camera,	
		GeoSAR	
Fixed wing (propeller engine)	Cessna 402	Camera	26,900
	Commander 690	LiDAR	19,400
	Cessna 208	Camera	
		LiDAR	25,000
	Cessna 206	Camera	
	DHC-6 Twin	Camera	15,700
	Otter 300	LIDAR	25,000
	Diamond	Camera	
	DA42	LiDAR	18,000
	Pilatus PC-6	Camera	
	Porter	Camera	25,000
	Piper Navajo	
		LiDAR	26,000
	Partenavia	Camera	
	P.68	LiDAR	19,200
	Vulcanair P68	Camera	
	Observer	Camera	18,000
Gyroplan	AutoGyro	LiDAR	10,000
	Cavalon	Camera	
Helicopter	Eurocopter	LiDAR	15,000
	AS350	Camera	
	Robinson R44	LiDAR	14,000
		Camera	
	Bell 206	LiDAR	13,000
		Camera	
	Schweizer	LiDAR5	13,000
	Camera		

**Table 7:** UAV platforms for RS.

Weight (kg)	Aircraft Power/Type	Manufacturer/Model	Flying Time (min)	Flying Speed (m/s)	RS Sensors
0.7	Fixed-wing/electric	senseFly/eBee RTK	40	11–25	Camera
6.1	Fixed-wing/electric	AeroVironment/Puma AE	210	23	Camera
6.0	Quadro copter/electric	Microdrones/MD4-1000	90	12	Camera/LiDAR
4.6–6.6	Hexacopter/electric	Aibotix/Aibot X6	30	14	Camera
5	Fixed-wing/electric	Trigger Composites/Pteryx	120	12.5–15	Camera
1.3	Quadrocopter/electric	DJI/Phantom 2	25	15	Camera
2.5	Fixed-wing/electric	Trimble/UX5	50	22	Camera
2.7	Fixed-wing/electric	Topcon/SIRIUS PRO	50	18	Camera
5.1–5.8	Fixed-wing/electric	Hawkeye UAV/AeroHawk	90	16.5–19.5	Camera
6.9–9.5	Hexacopter/electric	TRGS/Li-AIR	15	8	LiDAR
9.5	Octocopter/electric	Altus UAS/Delta X8	10–14	12	Camera/LiDAR
25	Octocopter/electric	Riegl/Ricopter	30	22	LiDAR/camera
77	Helicopter/gas	Aeroscout/Scout B1-100	90		LiDAR
90	Helicopter/gas	IGI/geocopter	120–180		Camera/LiDAR
9.2	Octocopter/electric	Altigator/OnyxStar FOX-C8 HD LiDAR	20		LiDAR
38	Fixed-wing/gas	American Aerospace/RS-16	720–960	33	Camera

**Table 8:** Comparison among existing PA applications.

PA	Edge	Data	Soil	Soil	Air	Air	Vegetation	Web	Mobile	Light	Wind
**Application**	**Computing**	**Analytic**	**Moisture**	**Temperature**	**Moisture**	**Temperature**	**Index**	**Services**	**Services**	**Intensity**	**Velocity**
[1]	N	Y	N	N	N	Y	Y	N	N	N	N
[4]	N	Y	N	N	N	N	N	Y	Y	N	N
[28]	N	Y	N	N	N	N	Y	N	N	N	N
[29]	N	Y	N	N	N	N	Y	N	N	N	N
[41]	N	N	Y	N	N	N	N	N	N	N	N
[52]	N	Y	N	N	N	N	Y	N	N	N	N
[55]	N	N	Y	N	Y	Y	N	N	Y	N	N
[56]	Y	N	Y	Y	Y	Y	N	N	N	Y	N
[57]	N	Y	Y	Y	Y	Y	N	N	N	Y	N
[58]	N	N	N	Y	N	N	N	N	N	N	N
[59]	N	Y	N	N	N	N	Y	N	N	N	N
[60]	Y	Y	Y	N	Y	Y	N	Y	Y	Y	N
[61]	Y	Y	Y	Y	Y	Y	N	Y	Y	Y	Y
[62]	N	Y	N	N	N	Y	N	Y	N	N	N
[63]	N	Y	Y	Y	Y	Y	N	Y	N	N	N
[64]	N	Y	Y	N	Y	Y	N	Y	N	N	N
[65]	N	Y	Y	Y	N	Y	N	N	N	N	N
[66]	N	Y	N	N	N	N	Y	N	N	N	N
[67]	N	Y	Y	Y	N	Y	Y	Y	Y	N	N
[68]	N	Y	N	N	N	N	Y	N	N	N	N
Proposed system	Y	Y	Y	Y	Y	Y	Y	Y	Y	N	N
